# Cysteinyl Leukotriene Receptor Antagonists Associated With a Decreased Incidence of Cancer: A Retrospective Cohort Study

**DOI:** 10.3389/fonc.2022.858855

**Published:** 2022-04-07

**Authors:** Ha Young Jang, In-Wha Kim, Jung Mi Oh

**Affiliations:** College of Pharmacy and Research Institute of Pharmaceutical Sciences, Seoul National University, Seoul, South Korea

**Keywords:** cysteinyl leukotriene receptor antagonists, cancer, cancer prevention, drug repurposing, observational study

## Abstract

**Aim:**

Cysteinyl leukotrienes receptor antagonists (LTRAs) are promising chemoprevention options to target cysteinyl leukotriene signaling in cancer. However, only a number of randomized clinical trials (RCTs) or observational studies have been conducted to date; thus, the effect of LTRAs on patients is yet to be elucidated. Using insurance claim data, we aimed to evaluate whether LTRAs have cancer preventive effects by observing patients who took LTRAs.

**Method:**

Patients diagnosed with asthma, allergic rhinitis, chronic cough, and have no history of cancer were followed-up from 2005 to 2017. Cox proportional hazard regression analysis was conducted to estimate the hazard ratios (HRs) for cancer risk of LTRA users.

**Result:**

We followed-up (median: 5.6 years) 188,906 matched patients (94,453 LTRA users and 94,453 non-users). LTRA use was associated with a decreased risk of cancer (adjusted HR [aHR] = 0.85, 95% confidence interval [CI] = 0.83–0.87). The cancer risk showed a tendency to decrease rapidly when LTRAs were used in high dose (aHR = 0.56, 95% CI = 0.40–0.79) or for longer durations of more than 3 years (aHR = 0.68, 95% CI = 0.60–0.76) and 5 years (aHR = 0.33, 95% CI = 0.26–0.42). The greater preventive effects of LTRAs were also observed in patients with specific risk factors related to sex, age, smoking, and the presence of comorbidities.

**Conclusion:**

In this study, we found that LTRA use was associated with a decreased risk of cancer. The high dose and long duration of the use of LTRAs correlated with a lower cancer risk. Since LTRAs are not yet used for the prevention or treatment of cancer, our findings could be used for developing a new chemo-regimen or designing feasible RCTs.

## Introduction

Cancer is the leading cause of death in Korea, and the mortality rate of this disease continuously to increase annually (1). As the cancer incidence continues to rise, the importance of cancer prevention is being emphasized. Cancer treatment is expensive as well as developing effective anticancer drugs. Thus, if cancer is successfully prevented, the overall medical cost can be reduced. Moreover, it is also challenging to plan cancer prevention strategies through clinical trials in terms of its duration and cost. Cancer prevention clinical trials take more than 5-10 years to complete and usually require thousands of participants. The estimated cost for large clinical trials involving more than 10,000 people is approximately $100 to $200 million (2). Despite decade-long efforts to find effective cure, candidates for anticancer drugs are usually discontinued during the phase 3 of the clinical trials due to problems, such as efficacy and toxicity (3). As the results of these trials do not always lead to successful cancer prevention strategies, there is an urgent need for identifying alternative drug therapies effective in preventing cancer. Drug repurposing is the process of searching for new indications for drugs that already exist in the market (4). Since this method is based on previously accumulated research and development data, the new drug development process can be accelerated, cutting costs at the same time (5). Recently, many studies on drug repurposing are being conducted based on genome, phenome, and insurance claim data (6).

Inflammation is a critical part in the pathogenesis of cancer, and the correlation of high levels of cysteinyl leukotrienes (CysLT) and CysLT1 receptor (CYsLTR) with various types of cancer have been reported several times in *in-vitro* studies (7–12). CYsLTR antagonists (LTRAs), including montelukast, pranlukast, and zafirlukast, have been widely used for treating asthma, allergic rhinitis, or chronic cough (13), and are the most promising chemoprevention options to target CysLT signaling in cancer. In addition to CysLT1 signaling, montelukast essentially induces apoptosis in cancer cells while zafirlukast is found to be involved in the cancer cell cycle (14, 15). Moreover, the role of LTRAs could also be associated with cancer metastasis, showing cell migration and invasion were suppressed in glioblastoma cells (14), colon cancer cells (16), skin cancer cells (17), and 5-FU-resistant colon cancer cells (18). However, the chemopreventive effects of LTRAs described above are all reported in *in-vitro* studies. Thus, it is still questionable whether the same effects can be observed in people taking LTRAs, especially since only limited randomized clinical trials (RCTs) and observational studies for humans are available (19). To observe the cancer-preventing effects of LTRAs in humans, a long-term follow-up study with a sufficiently large cohort size is essential. Therefore, using insurance claim data, we aimed to evaluate whether LTRAs have cancer prevention effects in a real-world setting by observing patients who took LTRAs.

## Materials and Methods

### Study Design and Sources

This study used a cohort study design and analyzed the health insurance data officially provided by the Korean National Health Insurance Service (KNHIS) (20). The insurance data included the patients’ demographic, diagnosis, procedure, and prescription data. Additionally, physical examination data that were linked to the KNHIS data were used. Physical examination information included the body mass index (BMI), smoking status, alcohol consumption, and exercise data. The requirement for the written informed consent from the participants was waived and all participants were anonymized by a randomized identification number. This study was approved by the institutional review board (IRB) of Seoul National University (IRB No. E1901/003-004).

### Study Population

To evaluate the effect of LTRA use on the prevention of cancer, patients diagnosed with asthma, allergic rhinitis, or chronic cough more than twice from 2005 to 2011 were included. Diagnosis of each disease was identified by the recorded diagnostic code of J45.x, J30.x, and R05.x for asthma, allergic rhinitis, and chronic cough in the claim, respectively. Patients who met the following criteria were excluded: diagnosed with asthma, allergic rhinitis, or chronic cough between 2002 and 2004; diagnosed with cancer before each patient’s index date; received LTRAs before being diagnosed with asthma, allergic rhinitis, or chronic cough; whose follow-up period is less than 1 year; whose day of LTRA use is less than 30 days.

### Ascertainment of Exposure

The LTRAs involved in this study include montelukast, pranlukast, and zafirlukast based on the anatomical therapeutic chemical (ATC) classification system. Information of the administered dose, frequency, and duration of the use of LTRAs were retrieved from the KNHIS database. Patients with no history of LTRA use were included in the non-user group. For the LTRA users, each daily dose was calculated by multiplying the number of tablets to be taken each day by the dose of each tablet, and this was converted to the defined daily dose (DDD), which is assigned by the World Health Organization’s Collaborating Center (WHOCC) for Drug Statistics Methodology (www.whocc.no/atc_ddd_index) (21). The cumulated dose was defined as the sum of multiplying the prescribed duration by the defined daily dose (DDD) of LTRAs.

### Ascertainment of Cancer

Individuals were followed-up until 2017, and outcomes were recorded from the individual’s index date. Primary endpoint of the study was cancer. Cancer event was defined based on the International Classification of Diseases-10 (ICD-10) codes (C00-C97). Cancer with the top 5 mortality rates (lung, hepatic, colorectal, stomach, pancreatic) and additional cancer types (breast, urological, skin, and brain/central nervous system cancer) were defined as secondary endpoints (1).

### Confounding Variables

Baseline characteristics, potentially influencing the study outcomes were included. These include demographic information, such as age at enrollment, sex, index year, region, and economic status. Region information was also collected by dividing the patients into special metropolitan city, metropolitan city, and province based on the patients’ insurance payment regions. Economic status of the enrolled participants was assessed based on income-related insurance payment. Concomitant asthma, anti-allergy medications, and initial diagnosis (asthma, allergic rhinitis, or chronic cough) within 1 year of index date were evaluated. Comorbidity burden was measured using the updated Charlson comorbidity index (CCI) to classify the level of comorbidity up to 1 year of index date (22). Furthermore, information on the smoking status and alcohol intake from questionnaire data and the BMI from physical examination data were collected.

### Statistical Analysis

Statistical analyses were performed for the intention-to-treat population. In the LTRA user group, if the date of LTRA initiation differed from the time of diagnosis, the patients would have periods during which cancer could not have been affected by treatment (immortal time). Therefore, each patient’s index date was defined as the very first date when LTRAs were prescribed for the LTRA users. The index date of non-users was then matched with the index date of the LTRA users. Patients were followed-up until the earliest onset of cancer, the date of the last follow-up, or the end of the study period. To adjust the effect of confounding variables between the LTRA user and non-user groups, propensity score matching was done. Propensity score was estimated by logistic regression with variables, including age, index year, region, economic status, co-medications, initial diagnosis, smoking status, alcohol intake, and BMI. LTRA users were matched 1:1 to non-users with the greedy 5 to 1 digit matching algorithm (23). Subsequently, the distribution of the propensity score before and after matching was inspected and the distribution of baseline covariates was evaluated with standardized difference. Standardized difference of over 0.1 was regarded as a sign of imbalance (24).

Cox proportional hazard regression was used to estimate the hazard ratio (HR) of LTRAs for cancer risk, with 95% confidence interval (CI). The confounding factors used were the age at enrollment, sex, index year, region, economic status, concomitant asthma/anti-allergy medications, initial diagnosis, CCI, smoking status, alcohol intake, and BMI. To test the robustness of our model, sensitivity analyses were performed. To prevent the LTRAs exposure factor from affecting the main outcomes, we applied a different exposure definition. In our original study design, the LTRAs exposure was defined as the sum of doses of the prescribed medications. In the sensitivity analysis, a new gap concept was defined to see the continuous use of LTRAs; if the gap between prescription refills was <30 days or at 50% of each prescription period, the patient was considered to have continued LTRA use. If the gap exceeded the predefined threshold, it was considered as patients have stopped and have not taken LTRAs any longer. Another sensitivity analysis was conducted by narrowing the index date between 2008 to 2011, and any changes in the risk of cancer were evaluated by calculating the HRs. Analyses were done with SAS software version 9.4 (SAS Institute Inc., Cary, NC, USA).

## Results

### Demographics

**Figure 1:**
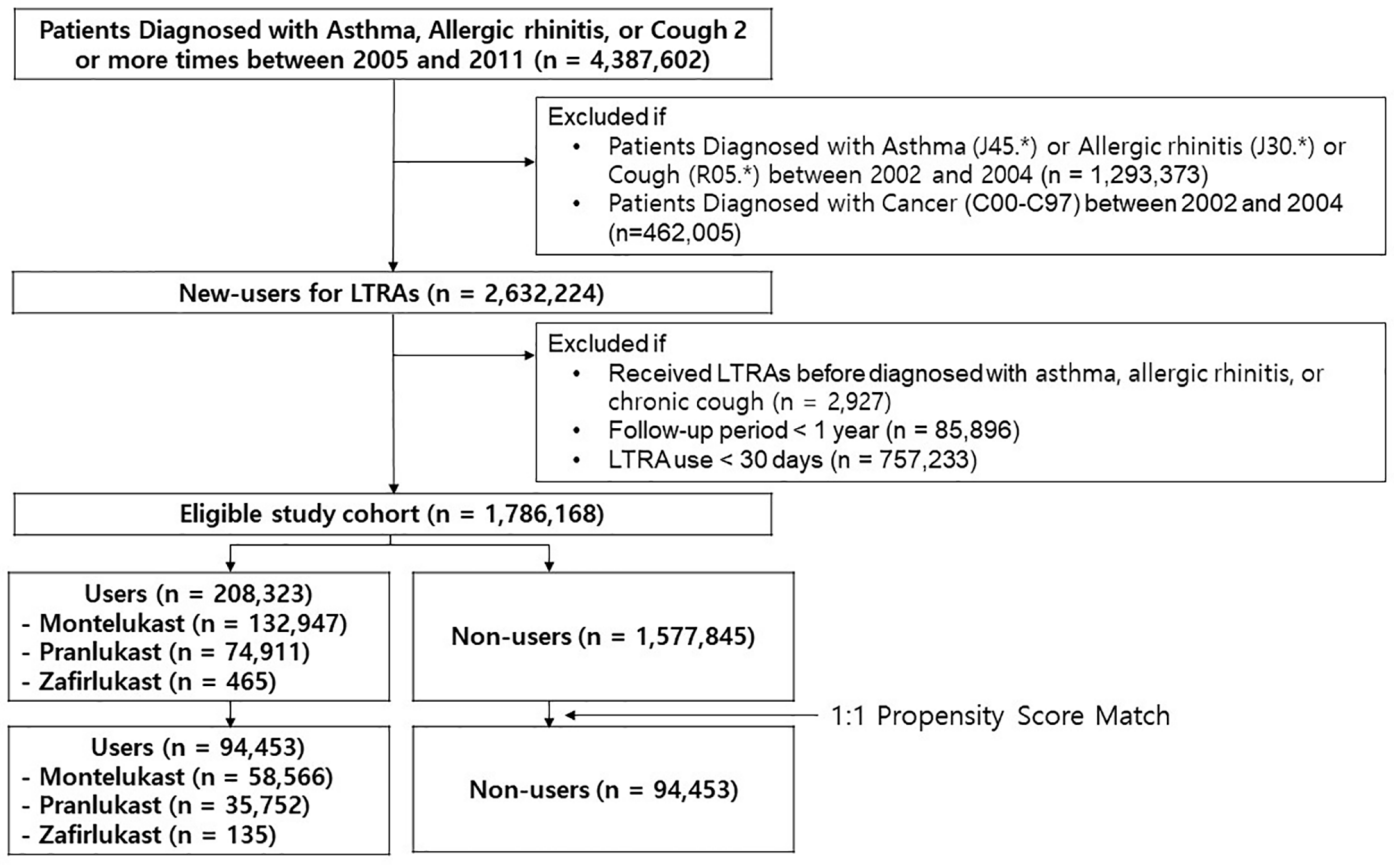
Study flow chart. LTRAs, Cysteinyl leukotrienes receptor antagonist.

Among all the patients diagnosed with asthma, allergic rhinitis, or chronic cough two or more times between 2005 and 2011 (n = 4,387,602), a total of 2,632,224 newly diagnosed patients with these conditions without a cancer history were identified (**Figure 1**). After excluding the patients who do not meet the predefined inclusion criteria, the eligible study cohort included 1,786,168 patients (208,323 LTRA users and 1,577,845 non-users). LTRA users took more co-medications and had higher CCI scores. The proportion of patients who were diagnosed with asthma was higher in LTRA users (82.2%) than non-users (53.3%). After the propensity score matching, 94,453 LTRA users were matched with 94,453 non-users. The above difference (co-medications, CCI, and initial diagnosis) was reduced, and standardized differences were below 0.1 for all covariates (**Table 1**). The median length of follow-up was 5.6 years (5.5 and 5.7 years for non-users and LTRA users, respectively). The median duration of LTRAs prescription during follow-up (65 days, interquartile range: 41-150 days) and mean age of patients [56.4 years; men: 42.6% (n = 80,533)] were shown. The most frequently used DDD were intermediate doses (64.1%), followed by low doses (35.5%), and high doses (0.4%).

**Table 1:** Baseline characteristics.

Characteristics	Non-users (N=94,453)	LTRAs users (N=94,453)	STD
Sex (male)	40,515 (42.8)	40,018 (42.4)	-0.005
Age (year)	51.4 ± 11.2	51.3 ± 12.4	0.014
BMI (kg/m^2^)	23.9 ± 3.2	23.9 ± 3.5	0.001
Drink (times/week)	0.9 ± 1.5	0.9 ± 1.5	-0.005
Economic status^a^			
1	10929 (11.6)	11045 (11.7)	0.029
2	14083 (14.9)	14201 (15.0)	
3	22333 (23.6)	22042 (23.3)	
4	23318 (24.7)	23474 (24.9)	
5	23790 (25.2)	23691 (25.1)	
Comorbidities			
Asthma	72014 (76.2)	72262 (76.5)	0.041
Allergic rhinitis	16085 (17.0)	15729 (16.7)	
Chronic cough	6354 (6.7)	6462 (6.8)	
Index year			
2008	8871 (9.4)	8978 (9.5)	0.051
2009	12961 (13.7)	12857 (13.6)	
2010	12806 (13.6)	12760 (13.5)	
2011	14097 (14.9)	14306 (15.1)	
2012	15933 (16.9)	15910 (16.8)	
2013	12605 (13.4)	12559 (13.3)	
2014	9938 (10.5)	10077 (10.7)	
2015	7242 (7.7)	7006 (7.4)	
Charlson comorbidity index			
0	7539 (8.0)	7360 (7.8)	0
1	25567 (27.1)	25633 (27.1)	
2	9370 (9.9)	9362 (9.9)	
3	51977 (55.0)	52098 (55.2)	
Smoking			
Never	66822 (70.8)	67058 (71.0)	0
History of smoking	8708 (9.2)	8518 (9.0)	
Current smoking	18923 (20.0)	18877 (20.0)	
Co-medications			
Xanthines	48267 (51.1)	47859 (50.7)	-0.009
β-Blockers	56710 (60.0)	56803 (60.1)	0.002
Anti-cholinergics	7874 (8.3)	7378 (7.8)	-0.019
Systemic steroids	79022 (83.7)	79608 (84.3)	0.017
Region			
Special metropolitan city	49025 (51.9)	49054 (51.9)	0.025
Metropolitan city	21916 (23.2)	21982 (23.3)	
Province	23512 (24.9)	23417 (24.8)	

Values are represented as mean ± standard deviation or number (%); LTRAs, Cysteinyl leukotrienes receptor antagonist; STD, standardized difference. ^a^Economic status was assessed based on income-related insurance payment; BMI, body mass index.

### Risk of Cancer in LTRAs Users

The median time of the first onset of cancer events was 3.4 years. The incidence rates of all recorded cancer types were shown in **Supplementary Table S1**. The use of LTRAs showed a significantly decreased risk of overall cancers (adjusted HR [aHR] = 0.85, 95% CI = 0.83–0.87). When examining each type of cancer, hepatic cancer (aHR = 0.73, 95% CI = 0.68–0.79), colorectal cancer (aHR = 0.83, 95% CI = 0.76–0.91), gastric cancer (aHR = 0.69, 95% CI = 0.62–0.76), breast cancer (aHR = 0.77, 95% CI = 0.71–0.83), and urological cancer (aHR = 0.92, 95% CI = 0.86–0.97) were significantly associated with LTRA use. In contrast, LTRAs showed no significant effect on lung, pancreatic, skin, and brain/central nervous system cancers (**Table 2**).

**Table 2:** Hazard ratios for each cancer components.

	Events	Person-year	Hazard ratio (95% CI)	
			Unadjusted	Adjusted
***All Cancer***				
Non-users	11369	520292	–	–
LTRAs	10399	536725	0.88 (0.86 – 0.91)	0.85 (0.83 – 0.87)
***Lung Cancer***				
Non-users	989	551209	–	–
LTRAs	1201	560047	1.19 (1.09 – 1.29)	1.06 (0.94 – 1.16)
***Liver Cancer***				
Non-users	1681	548448	–	–
LTRAs	1271	559331	0.74 (0.69 – 0.79)	0.73 (0.68 – 0.79)
***Colorectal Cancer***				
Non-users	1133	550157	–	–
LTRAs	1017	559934	0.88 (0.81 – 0.96)	0.83 (0.76 – 0.91)
***Stomach Cancer***				
Non-users	819	551045	–	–
LTRAs	625	560859	0.75 (0.67 – 0.83)	0.69 (0.62 – 0.76)
***Pancreas Cancer***				
Non-users	672	551969	–	–
LTRAs	641	561173	0.93 (0.84 – 1.04)	0.91 (0.81 – 1.01)
***Breast Cancer***				
Non-users	1433	549177	–	–
LTRAs	1181	559698	0.80 (0.74 – 0.87)	0.77 (0.71 – 0.83)
***Urological Cancer***				
Non-users	2146	547338	–	–
LTRAs	2191	557066	1.00 (0.94 – 1.06)	0.92 (0.86 – 0.97)
***Skin Cancer***				
Non-users	516	550132	–	–
LTRAs	534	558712	1.02 (0.90 – 1.15)	1.00 (0.88 – 1.14)
***Brain and Central Nervous System Cancer***				
Non-users	175	548347	–	–
LTRAs	150	554163	0.85 (0.68 – 1.05)	0.83 (0.67 – 1.03)

Hazard ratio was adjusted for age at enrollment, sex, index year, region, economic status, concomitant asthma/anti-allergy medications, initial diagnosis, charlson comorbidity index, smoking status, alcohol intake, and body mass index. CI, confidence interval; LTRAs, Cysteinyl leukotrienes receptor antagonist.

### Risk of Cancers by LTRAs Dose, Duration, and Cumulative Dose

When examining the cancer risk in terms of LTRA dose, the low (aHR = 0.89, 95% CI = 0.86–0.92) and intermediate doses (aHR = 0.86, 95% CI = 0.83–0.89) showed similar aHRs to the original results (**Table 3**). The aHR was also observed to be significantly lowered when the high dose was used (aHR = 0.56, 95% CI = 0.40–0.79). When the period of use of LTRAs was analyzed, the cancer risk showed a tendency to rapidly decrease when LTRAs were used for more than 3 years (aHR = 0.68, 95% CI = 0.60–0.76). Furthermore, the aHR decreased to 0.33 (95% CI = 0.26–0.42) when LTRA usage exceeds 5 years. A similar pattern was observed when the analysis was performed according to the cumulative dose obtained through the multiplication of dose and duration (cumulative DDD*year [cDY]). A significant decrease in aHR was shown when the cumulative dose was more than 5 cDY (aHR = 0.53, 95% CI = 0.47–0.60).

**Table 3:** Hazard ratios for cancer according to dose, duration, and cumulative dose of cysteinyl leukotriene receptor antagonists.

	Events	Person-years	Adjusted Hazard ratio (95% CI)
***Dose***			
Non-users	11369	520292	–
<0.5 DDD	4439	228313	0.89 (0.86 – 0.92)
0.5–1.0 DDD	5926	305943	0.86 (0.83 – 0.89)
≥1.0 DDD	34	2469	0.56 (0.40 – 0.79)
***Duration***			
Non-users	11369	520292	–
<0.5 year	7689	419707	0.85 (0.82 – 0.87)
0.5–1 year	1086	50909	0.87 (0.82 – 0.93)
1–3 year	1269	45512	1.02 (0.97 – 1.09)
3–5 year	281	13656	0.68 (0.60 – 0.76)
≥5 year	74	6941	0.33 (0.26 – 0.42)
***Cumulative dose***			
Non-users	11369	520292	–
<0.5 cDY	6854	376505	0.84 (0.81 – 0.87)
0.5–1 cDY	1426	68835	0.89 (0.84 – 0.94)
1–3 cDY	1352	56193	0.95 (0.89 – 1.00)
3–5 cDY	466	16221	1.03 (0.94 – 1.13)
≥5 cDY	301	18971	0.53 (0.47 – 0.60)

Hazard ratio was adjusted for age at enrollment, sex, index year, region, economic status, concomitant asthma/anti-allergy medications, initial diagnosis, Charlson comorbidity index, smoking status, alcohol intake, and body mass index. cDY, cumulative defined daily dose*year; CI, confidence interval; DDD, defined daily dose; LTRAs, Cysteinyl leukotrienes receptor antagonists.

### Sensitivity Analyses

The cancer prevention effect of LTRAs was the same after the gap change to 30 days and at 50% proportion of permissible gap. LTRA usage still significantly lowered the risk of cancer, and similar results were observed across all cancer types (lung, hepatic, colorectal, gastric, pancreatic, breast, urological, skin, and brain/central nervous system cancer) (**Supplementary Table S2**). In the analysis for cancer risk by narrowing the index period between 2008 to 2011, the same results for all cancer types were observed (**Supplementary Table S3**).

### Subgroup Analyses

**Figure 2:**
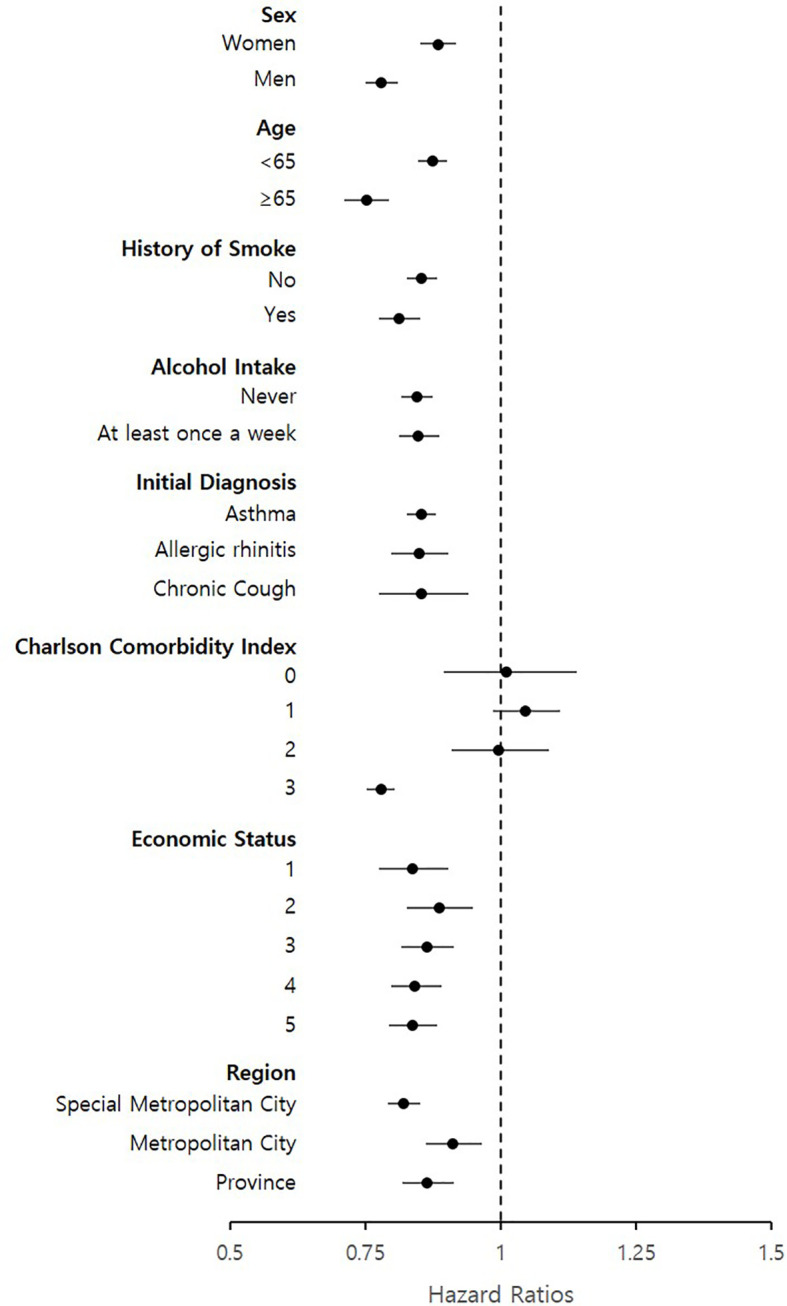
Subgroup analysis of hazard ratios for cancer events based on patient’s sex, age history of smoke, alcohol intake, initial diagnosis, charlson comorbidity index, economic status and region.

The greater preventive effects of LTRAs were observed in men (aHR = 0.78, 95% CI = 0.75–0.81), patients aged >65 years (aHR = 0.75, 95% CI = 0.71–0.79), and with a history of smoking or still currently smoking (aHR = 0.81, 95% CI = 0.78–0.85) compared to women, patients aged ≤65 years, and those who never smoked, respectively (**Figure 2**). LTRA use in patients with CCI scores of 0 showed no significant association with cancer (aHR = 1.01, 95% CI = 0.89–1.14); however, with higher CCI scores, the HR gradually decrease from 1.05 (95% CI 0.99–1.11) (CCI score: 1) to 0.78 (95% CI 0.75–0.80) (CCI score: 3). No significant differences in aHR were observed according to the patients’ alcohol intake, initial diagnosis, economic status, and region.

## Discussion

Our study analyzed patients who are using LTRAs through a long follow-up study. To our knowledge, this is the first research that consider the demographic information, co-medications, underlying comorbidities, and the patients’ physical examination data, including smoking status, alcohol intake, and BMI, while using a sufficiently large sample size. Our study results found that LTRA use was associated with an overall decreased risk of cancer. In addition, by dividing the dose and period of LTRA use into several subgroups, our study could identify the amount of dose and duration that may significantly lower the risk of cancer.

A previous cohort study also showed that the use of LTRAs significantly decreased the overall cancer risk, specifically for lung, colorectal, and breast cancer (25). The same trends were also found in our study; however, the magnitude of the reduced risk was smaller than the previous reported study. This result can be attributed to differences in the sample size and in the use of various covariates. In the work of Tsai et al., the number of patients after the propensity score matching was 25,110 (4,185 in the taking group, 20,925 in the non-taking group), which was much smaller than the 188,906 participants in our study. In addition, their study did not consider the variables related to lifestyle (e.g., smoking status, alcohol intake), which are major risk factors of cancer.

We found that the use of LTRAs had a significant preventive effect on overall cancers, which was consistent with other previous findings. Many studies have reported that LTRAs are effective not only for treatment (14, 15, 26, 27), including cancer metastasis (14, 16, 17, 28), but also for prevention (11, 25, 29–33), so it seems that LTRAs can be used in various stages of cancer. First, LTRAs inhibit the growth and/or induce apoptosis of a large series of human cancer cell lines. LTRAs inhibit growth of glioblastomas cells, by decreasing expression of B-cell lymphoma 2 (Bcl-2) protein and reducing the phosphorylation of extracellular signal-regulated kinase 1/2 (14). In breast cancer cells, apoptosis was also induced (15). A similar mechanism was found in colon cancer. In addition to significant reductions in cell proliferation, adhesion and colony formation, the induction of cell cycle arrest and apoptosis were observed in a dose-dependent manner (26). Montelukast induced down-regulation of Bcl-2, up-regulation of Bcl-2 homologous antagonist/killer, and nuclear translocation of apoptosis-inducing factors in lung cancer cells (27). Second, LTRAs could inhibit metastasis of cancer by preventing tumor cell migration through both cerebral and peripheral capillaries (14). Matrix metallopeptidase-9 (MMP-9) degrades extracellular matrix proteins and was increased in colon cancer patients. The MMP-9 expression and activity were reduced by montelukast (16). LTRAs inhibited epidermal growth factor-induced T cell lymphoma invasion and metastasis inducing protein 1 expression in skin cancer cells (17). There seems to be a difference in roles of preventive mechanisms within the LTRAs. Pranlukast can inhibit tumor cell migration through both the brain and peripheral capillaries, whereas montelukast inhibits tumor cell migration only in the peripheral capillaries (28). The preventive effect of LTRAs has been reported in several *in-vitro* and *in-vivo* studies for certain cancers, including colorectal (29), gastric (11), and pancreatic cancer (30). A previous cohort study also showed similar results, reporting that the risks of breast, colorectal, and liver cancers were significantly reduced (25). However, a non-significant association between lung cancer and LTRA use was found in our study, while other groups have reported its cancer risk reduction effect (25, 31). Three studies also showed that LTRAs reduced the risk of metastatic lung cancer, but not of lung cancer itself (28, 32, 33).

Despite these efforts, there have not been reports on any definite association between LTRAs and a specific type of cancer yet. The pathogenesis of cancer appears to be multifactorial, and such findings may have arisen due to differences in the study samples, study designs, or statistical methods. Tsai et al. (2016) also showed that the use of LTRAs was an independent protecting factor for overall cancers, reporting an HR of 0.31 (95% CI: 0.24–0.39). The magnitude of reduced risk was found to be smaller in our study (HR 0.85, 95% CI = 0.83–0.87), which might be due to larger sample size and the use of additional covariates. For instance, the patient’s smoking status had a high HR range, 1.16 (against liver cancer) to 1.67 (against lung cancer), implying that the smoking covariate is a large proportion in our cox proportional hazard regression model.

In our study, the analysis of dose and duration of LTRAs use is noteworthy. Most LTRA prescriptions (99.6%) provided for the patients in this study were low (<0.5 DDD) or intermediate (0.5 ≤ DDD < 1.0), and only a few proportions were high (0.4%). Our results showed that overall cancer risk was rapidly lowered when LTRAs were used in high doses. In the duration analysis, >3 years of LTRA use correlated with a much lower HR for cancers. LTRAs are usually considered as safe during long-term administration even at doses substantially higher than the recommended dose (34). Therefore, this suggests that future studies should consider a higher dose and longer duration when prescribing LTRAs to be able to secure its anti-cancer property without having to worry about its side effects. However, recently, neuropsychiatric events were reported in post-marketing surveillance and resulted in safety alert in 2008 and a black box warning in 2020. Additionally, conflicting reports on the association between LTRAs and neuropsychiatric events have been published (35, 36). Therefore, it is necessary to pay attention to these precautions. The results of our study could also be used in the design of clinical trials. For instance, RCTs have been conducted with zileuton, a 5-lipoxygenase inhibitor that shares a similar mechanism with LTRAs, as an adjuvant agent to conventional chemotherapy for lung cancer patients (37). With this, new and improved RCTs can be conducted using LTRAs as an addition to existing anticancer therapies.

In our subgroup analysis, notable results were also observed in specific patient groups. The greater preventive effects of LTRAs in lowering the risk of cancer were observed in the following: in men, patients aged >65 years, patients with a history of smoking or are currently smoking, and those with high CCI scores. Considering that men, aged patients, smoking, and the presence of various comorbidities are well-known risk factors, LTRAs may contribute to lowering the cancer risks in patients with these particular characteristics. For the design of realistic and feasible clinical trials, the selection of specific patient groups with the above-mentioned risk factors may be beneficial and more effective.

There are several limitations encountered in our study. Due to the nature of the real-world data, the purpose of prescribing LTRAs to the patients was not for cancer prevention. Moreover, our study does not include an active comparator, and therefore it may be susceptible to selection bias. However, to reduce bias, as many variables were collected and matched to minimize the differences between groups. But note that there may still be some residual confounding after bias reduction. It was impossible to specify the stage/subtype of cancer because the disease information provided by the ICD-10 code was limited. We also suggest that some caution should be exercised when interpreting our results. There have been several studies showing that the use of LTRAs are also effective in reducing the risk of lung cancer, but the results in our study were not statistically significant (25, 31). Considering that baseline comorbidities, such as asthma, can have a significant effect on the occurrence of lung cancer (38, 39), this study may not have completely ruled out the effects of other comorbid diseases on cancer because it used CCI score as an indirect measure of various disease severity. Likewise, our study used a retrospective cohort design and not all information are included and available in the KNHIS data. Therefore, although we adjusted for all possible confounders, there still might be residual confounding factors present during our analyses.

The findings of our study suggest that the use of LTRAs was associated with a decreased risk of overall cancer. The high dose and long duration of LTRA use correlated with the lowered risk. The greater preventive effects of LTRAs were also observed in patients with specific risk factors related to sex, age, smoking, and the presence of comorbidities. As LTRAs have not yet been used for the prevention or treatment of cancer, our findings could be used for developing a new chemo-regimen or in designing feasible RCTs. For future studies, further research is needed to elucidate the specific mechanism and clinical significance of our results.

## Data Availability Statement

The data analyzed in this study is subject to the following licenses/restrictions: Data that can view all the records of a patient are difficult to share due to the policy of the NHIS. It can only be viewed in anonymized form when analyzed. Therefore, if there is a request for original data, the statistical data obtained after the desired statistical processing on the server will be shared. Requests to access these datasets should be directed to National Health Insurance Service, nhiss.nhis.or.kr.

## Ethics Statement

The studies involving human participants were reviewed and approved by Institutional review board (IRB) of Seoul National University (IRB No. E1901/003-004). Written informed consent for participation was not required for this study in accordance with the national legislation and the institutional requirements.

## Author Contributions

HJ contributed to the conception and design of the study, data acquisition, analysis and interpretation of results, drafting, and revision of the manuscript. JO and I-WK contributed to the conception and design of the study, analysis and interpretation of results, and revision of the manuscript. All authors contributed to the article and approved the submitted version.

## Funding

This study was supported by the National Research Foundation of Korea grant funded by the Korean government (MSIT) (no. NRF-2018R1A2B6001859).

## Conflict of Interest

The authors declare that the research was conducted in the absence of any commercial or financial relationships that could be construed as a potential conflict of interest.

## Publisher’s Note

All claims expressed in this article are solely those of the authors and do not necessarily represent those of their affiliated organizations, or those of the publisher, the editors and the reviewers. Any product that may be evaluated in this article, or claim that may be made by its manufacturer, is not guaranteed or endorsed by the publisher.

## References

[1] Korea VSDS Shin H-Y Kim J Lee S Park MS Park S. Cause-Of-Death Statistics in 2018 in the Republic of Korea. J Korean Med Assoc (2020) 63(5):286–97. doi: 10.5124/jkma.2020.63.5.286

[2] Guess HA Rudnick SA. Use of Cost-Effectiveness Analysis in Planning Cancer Chemoprophylaxis Trials. Control Clin Trials (1983) 4(2):89–100. doi: 10.1016/s0197-2456(83)80016-6 6411430

[3] Williams R. Discontinued in 2013: Oncology Drugs. Expert Opin Investig Drugs (2015) 24(1):95–110. doi: 10.1517/13543784.2015.971154 25315907

[4] Langedijk J Mantel-Teeuwisse AK Slijkerman DS Schutjens MH. Drug Repositioning and Repurposing: Terminology and Definitions in Literature. Drug Discov Today (2015) 20(8):1027–34. doi: 10.1016/j.drudis.2015.05.001 25975957

[5] Ashburn TT Thor KB. Drug Repositioning: Identifying and Developing New Uses for Existing Drugs. Nat Rev Drug Discov (2004) 3(8):673–83. doi: 10.1038/nrd1468 15286734

[6] Li J Zheng S Chen B Butte AJ Swamidass SJ Lu Z. A Survey of Current Trends in Computational Drug Repositioning. Brief Bioinform (2016) 17(1):2–12. doi: 10.1093/bib/bbv020 25832646 PMC4719067

[7] Zhang WP Hu H Zhang L Ding W Yao HT Chen KD. Expression of Cysteinyl Leukotriene Receptor 1 in Human Traumatic Brain Injury and Brain Tumors. Neurosci Lett (2004) 363(3):247–51. doi: 10.1016/j.neulet.2004.03.088 15182953

[8] Magnusson C Mezhybovska M Lorinc E Fernebro E Nilbert M Sjolander A. Low Expression of CysLT1R and High Expression of CysLT2R Mediate Good Prognosis in Colorectal Cancer. Eur J Cancer (2010) 46(4):826–35. doi: 10.1016/j.ejca.2009.12.022 20064706

[9] Matsuyama M Yoshimura R. Cysteinyl-Leukotriene1 Receptor is a Potent Target for the Prevention and Treatment of Human Urological Cancer. Mol Med Rep (2010) 3(2):245–51. doi: 10.3892/mmr_00000247 21472229

[10] Magnusson C Liu J Ehrnstrom R Manjer J Jirstrom K Andersson T. Cysteinyl Leukotriene Receptor Expression Pattern Affects Migration of Breast Cancer Cells and Survival of Breast Cancer Patients. Int J Cancer (2011) 129(1):9–22. doi: 10.1002/ijc.25648 20824707

[11] Venerito M Kuester D Harms C Schubert D Wex T Malfertheiner P. Upregulation of Leukotriene Receptors in Gastric Cancer. Cancers (Basel) (2011) 3(3):3156–68. doi: 10.3390/cancers3033156 PMC3759191 24212950

[12] Slater K Heeran AB Garcia-Mulero S Kalirai H Sanz-Pamplona R Rahman A. High Cysteinyl Leukotriene Receptor 1 Expression Correlates With Poor Survival of Uveal Melanoma Patients and Cognate Antagonist Drugs Modulate the Growth, Cancer Secretome, and Metabolism of Uveal Melanoma Cells. Cancers (Basel) (2020) 12(10):2950. doi: 10.3390/cancers12102950 PMC7600582 33066024

[13] Yokomizo T Nakamura M Shimizu T. Leukotriene Receptors as Potential Therapeutic Targets. J Clin Invest (2018) 128(7):2691–701. doi: 10.1172/JCI97946 PMC6025999 29757196

[14] Piromkraipak P Parakaw T Phuagkhaopong S Srihirun S Chongthammakun S Chaithirayanon K. Cysteinyl Leukotriene Receptor Antagonists Induce Apoptosis and Inhibit Proliferation of Human Glioblastoma Cells by Downregulating B-Cell Lymphoma 2 and Inducing Cell Cycle Arrest. Can J Physiol Pharmacol (2018) 96(8):798–806. doi: 10.1139/cjpp-2017-0757 29726704

[15] Suknuntha K Yubolphan R Krueaprasertkul K Srihirun S Sibmooh N Vivithanaporn P. Leukotriene Receptor Antagonists Inhibit Mitogenic Activity in Triple Negative Breast Cancer Cells. Asian Pac J Cancer Prev (2018) 19(3):833–7. doi: 10.22034/APJCP.2018.19.3.833 PMC5980863 29582642

[16] Vinnakota K Zhang Y Selvanesan BC Topi G Salim T Sand-Dejmek J. M2-Like Macrophages Induce Colon Cancer Cell Invasion *via* Matrix Metalloproteinases. J Cell Physiol (2017) 232(12):3468–80. doi: 10.1002/jcp.25808 28098359

[17] Magi S Takemoto Y Kobayashi H Kasamatsu M Akita T Tanaka A. 5-Lipoxygenase and Cysteinyl Leukotriene Receptor 1 Regulate Epidermal Growth Factor-Induced Cell Migration Through Tiam1 Upregulation and Rac1 Activation. Cancer Sci (2014) 105(3):290–6. doi: 10.1111/cas.12340 PMC4317946 24350867

[18] Satapathy SR Sjolander A. Cysteinyl Leukotriene Receptor 1 Promotes 5-Fluorouracil Resistance and Resistance-Derived Stemness in Colon Cancer Cells. Cancer Lett (2020) 488:50–62. doi: 10.1016/j.canlet.2020.05.023 32474153

[19] Saier L Peyruchaud O. Emerging Role of Cysteinyl LTs in Cancer. Br J Pharmacol (2021) 1–20. doi: 10.1111/bph.15402 33527344

[20] Cheol Seong S Kim YY Khang YH Heon Park J Kang HJ Lee H. Data Resource Profile: The National Health Information Database of the National Health Insurance Service in South Korea. Int J Epidemiol (2017) 46(3):799–800. doi: 10.1093/ije/dyw253 27794523 PMC5837262

[21] WHOCC. ATC Classifcation Index With DDDs. Oslo, Norway: WHO Collaborating Centre for Drug Statistics Methodology (2016). Available at: www.whocc.no/atc_ddd_index.

[22] Quan H Li B Couris CM Fushimi K Graham P Hider P. Updating and Validating the Charlson Comorbidity Index and Score for Risk Adjustment in Hospital Discharge Abstracts Using Data From 6 Countries. Am J Epidemiol (2011) 173(6):676–82. doi: 10.1093/aje/kwq433 21330339

[23] Parsons LS. Reducing Bias in a Propensity Score Matched-Pair Sample Using Greedy Matching Techniques (2001). Available at: http://www2.sas.com/proceedings/sugi26/p214-26.pdf (Accessed 2017-01-01).

[24] Zhang Z Kim HJ Lonjon G Zhu Y written on behalf of, A.M.E. Big-Data Clinical Trial Collaborative Group. Balance Diagnostics After Propensity Score Matching. Ann Transl Med (2019) 7(1):16. doi: 10.21037/atm.2018.12.10 30788363 PMC6351359

[25] Tsai MJ Wu PH Sheu CC Hsu YL Chang WA Hung JY. Cysteinyl 26. Leukotriene Receptor Antagonists Decrease Cancer Risk in Asthma Patients. Sci Rep (2016) 6:23979. doi: 10.1038/srep23979 27052782 PMC4823742

[26] Savari S Liu M Zhang Y Sime W Sjolander A. CysLT(1)R Antagonists Inhibit Tumor Growth in a Xenograft Model of Colon Cancer. PloS One (2013) 8(9):e73466. doi: 10.1371/journal.pone.0073466 24039952 PMC3764114

[27] Tsai MJ Chang WA Tsai PH Wu CY Ho YW Yen MC. Montelukast Induces Apoptosis-Inducing Factor-Mediated Cell Death of Lung Cancer Cells. Int J Mol Sci (2017) 18(7):1353. doi: 10.3390/ijms18071353 PMC5535846 28672809

[28] Nozaki M Yoshikawa M Ishitani K Kobayashi H Houkin K Imai K. Cysteinyl Leukotriene Receptor Antagonists Inhibit Tumor Metastasis by Inhibiting Capillary Permeability. Keio J Med (2010) 59(1):10–8. doi: 10.2302/kjm.59.10 PMC3397318 20375653

[29] Burke L Butler CT Murphy A Moran B Gallagher WM O’Sullivan J. Evaluation of Cysteinyl Leukotriene Signaling as a Therapeutic Target for Colorectal Cancer. Front Cell Dev Biol (2016) 4:103. doi: 10.3389/fcell.2016.00103 27709113 PMC5030284

[30] Hennig R Ding XZ Tong WG Schneider MB Standop J Friess H. 5-Lipoxygenase and Leukotriene B(4) Receptor Are Expressed in Human Pancreatic Cancers But Not in Pancreatic Ducts in Normal Tissue. Am J Pathol (2002) 161(2):421–8. doi: 10.1016/S0002-9440(10)64198-3 PMC1850753 12163367

[31] Kahnt AS Rorsch F Diehl O Hofmann B Lehmann C Steinbrink SD. Cysteinyl Leukotriene-Receptor-1 Antagonists Interfere With PGE2 Synthesis by Inhibiting mPGES-1 Activity. Biochem Pharmacol (2013) 86(2):286–96. doi: 10.1016/j.bcp.2013.05.005 23684692

[32] Lee KS Kim SR Park HS Jin GY Lee YC. Cysteinyl Leukotriene Receptor Antagonist Regulates Vascular Permeability by Reducing Vascular Endothelial Growth Factor Expression. J Allergy Clin Immunol (2004) 114(5):1093–9. doi: 10.1016/j.jaci.2004.07.039.32 15536415

[33] Gunning WT Kramer PM Steele VE Pereira MA. Chemoprevention by Lipoxygenase and Leukotriene Pathway Inhibitors of Vinyl Carbamate-Induced Lung Tumors in Mice. Cancer Res (2002) 62(15):4199–201. 12154018

[34] Gulati A. Review of the Role of Anti Leukotrienes in the Therapy of Allergic Rhinitis in Children (2013). Available at: https://www.who.int/selection_medicines/committees/expert/19/applications/LTRA_28_C_Ad.pdf (Accessed 2021.05.24).

[35] Umetsu R Tanaka M Nakayama Y Kato Y Ueda N Nishibata Y. Neuropsychiatric Adverse Events of Montelukast: An Analysis of Real-World Datasets and Drug-Gene Interaction Network. Front Pharmacol (2021) 12:764279. doi: 10.3389/fphar.2021.764279 34987393 PMC8720925

[36] Fox CW Khaw CL Gerke AK Lund BC. Montelukast and Neuropsychiatric Events - A Sequence Symmetry Analysis. J Asthma (2021) 1–7. doi: 10.1080/02770903.2021.2018705 34979844

[37] Edelman M. Carboplatin and Gemcitabine Combined With Celecoxib and/or Zileuton in Treating Patients With Advanced Non-Small Cell Lung Cancer (2003). Available at: https://clinicaltrials.gov/ct2/show/NCT00070486 (Accessed 2021-05-24).

[38] Koh WP Yuan JM Wang R Seow A Lee HP Yu MC. Chronic Rhinosinusitis and Risk of Lung Cancer in the Singapore Chinese Health Study. Int J Cancer (2008) 123(6):1398–402. doi: 10.1002/ijc.23623 PMC2728457 18548585

[39] Qu YL Liu J Zhang LX Wu CM Chu AJ Wen BL. Asthma and the Risk of Lung Cancer: A Meta-Analysis. Oncotarget (2017) 8(7):11614–20. doi: 10.18632/oncotarget.14595 PMC5355290 28086224

