# Supplementary material for: Cysteinyl Leukotriene Receptor Antagonists Associated With a Decreased Incidence of Cancer: A Retrospective Cohort Study
Source: Front Oncol. 2022 Apr 7;12:858855. doi: 10.3389/fonc.2022.858855 (PMC9021999; doi:10.3389/fonc.2022.858855)
Supplement: Supplementary file 1 [file Table_1.docx]

**Supplementary Table S1** Overall incidence of cancer

|  | **Events** | **Incidence rate (%)** |
| --- | --- | --- |
|  |  |  |
| ***All Cancer*** | 21768 | 11.5 |
| ***Lung Cancer*** | 2190 | 1.2 |
| ***Liver Cancer*** | 2952 | 1.6 |
| ***Colorectal Cancer*** | 2150 | 1.1 |
| ***Stomach Cancer*** | 1444 | 0.8 |
| ***Pancreas Cancer*** | 1313 | 0.7 |
| ***Breast Cancer*** | 2614 | 1.4 |
| ***Urological Cancer*** | 4337 | 2.3 |
| ***Skin Cancer*** | 1050 | 0.6 |
| ***Brain and Central Nervous System Cancer*** | 325 | 0.2 |

**Supplementary Table S2** Sensitivity Analysis 1

|  | **Events** | **Person-year** | **Hazard ratio (95% CI)** | |
| --- | --- | --- | --- | --- |
|  |  |  | **Unadjusted** | **Adjusted** |
| ***Permissive 30 days of gap*** | | | | |
| ***All Cancer*** | | | | |
| Non-users | 5424 | 235619 | - | - |
| LTRAs | 4763 | 241407 | 0.85 (0.82 – 0.89) | 0.82 (0.79 – 0.85) |
| ***Lung Cancer*** | | | | |
| Non-users | 506 | 249535 | - | - |
| LTRAs | 618 | 251407 | 1.21 (1.08 – 1.36) | 1.10 (0.97 – 1.24) |
| ***Liver Cancer*** | | | | |
| Non-users | 791 | 248377 | - | - |
| LTRAs | 573 | 251227 | 0.71 (0.64 – 0.79) | 0.70 (0.63 – 0.78) |
| ***Colorectal Cancer*** | | | | |
| Non-users | 540 | 249051 | - | - |
| LTRAs | 488 | 251414 | 0.89 (0.79 – 1.01) | 0.84 (0.75 – 0.95) |
| ***Stomach Cancer*** | | | | |
| Non-users | 392 | 249395 | - | - |
| LTRAs | 306 | 251851 | 0.77 (0.67 – 0.90) | 0.71 (0.61 – 0.83) |
| ***Pancreas Cancer*** | | | | |
| Non-users | 315 | 249909 | - | - |
| LTRAs | 303 | 251972 | 0.95 (0.81 – 1.12) | 0.93 (0.80 – 1.10) |
| ***Breast Cancer*** | | | | |
| Non-users | 546 | 249000 | - | - |
| LTRAs | 482 | 251530 | 0.87 (0.77 – 0.98) | 0.85 (0.75 – 0.96) |
| ***Urological Cancer*** | | | | |
| Non-users | 1100 | 247492 | - | - |
| LTRAs | 1044 | 250116 | 0.94 (0.86 – 1.02) | 0.86 (0.79 – 0.94) |
| ***Skin Cancer*** | | | | |
| Non-users | 274 | 248434 | - | - |
| LTRAs | 280 | 251271 | 1.01 (0.86 – 1.20) | 0.97 (0.82 – 1.16) |
| ***Brain and Central Nervous System Cancer*** | | | | |
| Non-users | 82 | 249132 | - | - |
| LTRAs | 72 | 251672 | 0.87 (0.63 – 1.20) | 0.82 (0.57 – 1.15) |
| ***Permissive 50% proportion of gap*** | | | | |
| ***All Cancer*** | | | | |
| Non-users | 8443 | 299168 | - | - |
| LTRAs | 5095 | 265057 | 0.68 (0.65 – 0.7) | 0.79 (0.76 – 0.82) |
| ***Lung Cancer*** |  |  |  |  |
| Non-users | 1038 | 323451 | - | - |
| LTRAs | 623 | 277197 | 0.68 (0.62 – 0.76) | 0.98 (0.88 – 1.09) |
| ***Liver Cancer*** |  |  |  |  |
| Non-users | 1156 | 322285 | - | - |
| LTRAs | 603 | 276916 | 0.61 (0.55 – 0.68) | 0.67 (0.60 – 0.74) |
| ***Colorectal Cancer*** |  |  |  |  |
| Non-users | 915 | 323005 | - | - |
| LTRAs | 515 | 277150 | 0.65 (0.58 – 0.72) | 0.75 (0.67 – 0.84) |
| ***Stomach Cancer*** |  |  |  |  |
| Non-users | 687 | 323696 | - | - |
| LTRAs | 334 | 277538 | 0.56 (0.49 – 0.64) | 0.68 (0.59 – 0.77) |
| ***Pancreas Cancer*** |  |  |  |  |
| Non-users | 446 | 324898 | - | - |
| LTRAs | 311 | 277848 | 0.81 (0.70 – 0.94) | 0.87 (0.75 – 1.02) |
| ***Breast Cancer*** | | | | |
| Non-users | 628 | 323942 | - | - |
| LTRAs | 539 | 277142 | 1.02 (0.91 – 1.15) | 0.85 (0.75 – 0.96) |
| ***Urological Cancer*** | | | | |
| Non-users | 1794 | 320081 | - | - |
| LTRAs | 1099 | 275622 | 0.71 (0.66 – 0.77) | 0.84 (0.77 – 0.91) |
| ***Skin Cancer*** | | | | |
| Non-users | 335 | 321724 | - | - |
| LTRAs | 288 | 276842 | 1.00 (0.85 – 1.17) | 1.02 (0.87 – 1.18) |
| ***Brain and Central Nervous System Cancer*** | | | | |
| Non-users | 100 | 322471 | - | - |
| LTRAs | 71 | 275831 | 0.83 (0.61 – 1.13) | 0.86 (0.64 – 1.16) |

Hazard ratio was adjusted for age at enrollment, sex, index year, region, economic status, concomitant asthma/anti-allergy medications, initial diagnosis, Charlson comorbidity index, smoking status, alcohol intake, and body mass index. CI, confidence interval; LTRAs, Cysteinyl leukotrienes receptor antagonists

|  | **Events** | **Person-year** | **Hazard ratio (95% CI)** | |
| --- | --- | --- | --- | --- |
|  |  |  | **Unadjusted** | **Adjusted** |
| ***Narrowing the Index period to 2008 - 2011*** | | | | |
| ***All Cancer*** | | | | |
| Non-users | 7382 | 337749 | - | - |
| LTRAs | 6753 | 349752 | 0.88 (0.85 – 0.91) | 0.81(0.79 – 0.84) |
| ***Lung Cancer*** | | | | |
| Non-users | 633 | 361330 | - | - |
| LTRAs | 851 | 367517 | 1.31 (1.12 – 1.46) | 1.07 (0.96 – 1.19) |
| ***Liver Cancer*** | | | | |
| Non-users | 1076 | 359269 | - | - |
| LTRAs | 824 | 367306 | 0.75 (0.68 – 0.82) | 0.73 (0.66 – 0.80) |
| ***Colorectal Cancer*** | | | | |
| Non-users | 734 | 360576 | - | - |
| LTRAs | 681 | 367728 | 0.91 (0.82 – 1.01) | 0.82 (0.73 – 0.91) |
| ***Stomach Cancer*** | | | | |
| Non-users | 537 | 361225 | - | - |
| LTRAs | 429 | 368412 | 0.78 (0.69 – 0.89) | 0.68 (0.59 – 0.77) |
| ***Pancreas Cancer*** | | | | |
| Non-users | 417 | 362037 | - | - |
| LTRAs | 404 | 368767 | 0.94 (0.82 – 1.08) | 0.89 (0.77 – 1.02) |
| ***Breast Cancer*** | | | | |
| Non-users | 991 | 359628 | - | - |
| LTRAs | 779 | 367541 | 0.76 (0.69 – 0.84) | 0.78 (0.71 – 0.86) |
| ***Urological Cancer*** | | | | |
| Non-users | 1314 | 358634 | - | - |
| LTRAs | 1381 | 365701 | 1.02 (0.95 – 1.11) | 0.88 (0.81 – 0.95) |
| ***Skin Cancer*** | | | | |
| Non-users | 364 | 358912 | - | - |
| LTRAs | 375 | 366711 | 1.01 (0.87 – 1.16) | 0.95 (0.81 – 1.10) |
| ***Brain and Central Nervous System Cancer*** | | | | |
| Non-users | 109 | 359121 | - | - |
| LTRAs | 95 | 366421 | 0.85 (0.65 – 1.12) | 0.83 (0.63 – 1.11) |

**Supplementary Table S3** Sensitivity Analysis 2

Hazard ratio was adjusted for age at enrollment, sex, index year, region, economic status, concomitant asthma/anti-allergy medications, initial diagnosis, Charlson comorbidity index, smoking status, alcohol intake, and body mass index. CI, confidence interval; LTRAs, Cysteinyl leukotrienes receptor antagonists
